# Molecular Dynamics Simulations Reveal Structural Interconnections within Sec14-PH Bipartite Domain from Human Neurofibromin

**DOI:** 10.3390/ijms23105707

**Published:** 2022-05-20

**Authors:** Fabio Rizza, Jacopo Vertemara, Renata Tisi

**Affiliations:** 1 Department of Biotechnology and Biosciences, University of Milano-Bicocca, 20126 Milan, Italy; fabio.rizza@unimib.it (F.R.); jacopo.vertemara@unimib.it (J.V.); 2 Milan Center for Neuroscience (NeuroMI), University of Milano-Bicocca, 20126 Milano, Italy

**Keywords:** MD, NF1, neuropathy, cancer-prone syndrome

## Abstract

Neurofibromin, the main RasGAP in the nervous system, is a 2818 aa protein with several poorly characterized functional domains. Mutations in the NF1-encoding gene lead to an autosomal dominant syndrome, neurofibromatosis, with an incidence of 1 out of 3000 newborns. Missense mutations spread in the Sec14-PH-encoding sequences as well. Structural data could not highlight the defect in mutant Sec14-PH functionality. By performing molecular dynamics simulations at different temperatures, we found that the lid-lock is fundamental for the structural interdependence of the NF1 bipartite Sec14-PH domain. In fact, increased flexibility in the lid-lock loop, observed for the K1750Δ mutant, leads to disconnection of the two subdomains and can affect the stability of the Sec14 subdomain.

## 1. Introduction

Neurofibromatosis type 1 (NF-1) is an autosomal dominant disease with an incidence of 1 in over 3000 births arising from mutations in the neurofibromin-encoding gene, *NF1*. Affected patients manifest typical symptoms, such as cafe-au-lait spots on the skin, and are likely to develop tumors in the peripheral nervous system and suffer from learning disabilities [1].

Neurofibromin (NF1) is a 2818 residue protein, mainly expressed in the brain, whose pivotal function is to act as a GAP (GTPase-activating protein) in order to quench growth signals [2]. This function is carried out by a 300-residue domain located in a central region of the sequence and called GRD (GAP-related domain), which provides a binding groove for Ras and a typical arginine finger that assists GTP hydrolysis in the active site of Ras [3]. Apart from the catalytic GRD, neurofibromin is a poorly understood protein. There are several known cellular partners, but the biological meaning and molecular features of most interactions have not been elucidated yet [4,5]. Additionally, NF1-associated mutations are widespread in the gene rather than focused in the GAP-domain-encoding region [6].

Very little information is available about neurofibromin structural features. The only structurally investigated domains up to date are the GRD and the Sec14-PH domains, located just downstream of the GRD, due to the difficulty in purifying other portions of the protein [7].

The X-ray structure determination of the bipartite Sec14-PH, which hosts many of the NF1-associated mutations, was unfortunately not resolutive to understanding the mechanism at the base of the pathogenicity of the investigated mutations [8]. This region consists of two subdomains, structurally similar to two well-known folds, a Sec14 and a PH module. The first Sec14-containing protein was characterized in yeast as a lipid exchanger between membranes. Many proteins have hitherto been characterized in eukaryotes that possess a Sec14 module [9]. It consists of a globular fold with a lipid cage that is gated by an alpha-helix (also referred to as the lid helix), which can undergo an extended conformational change, as it is suggested by both crystal structures and simulations [10,11]. The PH domain is a typical protein module involved in signal transduction. PH domains from different proteins were reported to either bind lipids or mediate protein–protein interactions [12].

In the context of neurofibromin, the Sec14 and the PH domain are fused in a unique 250-residue structure. Its main feature is the structural interaction between the two parts. A long-loop protrusion (also called lid-lock), shaped as a beta-hairpin in the PH module, bends upon the lid helix in the Sec14, forcing it to adopt a closed conformation with a lipid molecule stuck inside [8]. Since the NF1 Sec14-PH domain can actually exchange lipids in vitro, its unique structure raises the question of the mechanism of the process [13]. Indeed, for it to achieve this task, an extended movement of the beta protrusion has been proposed for a concerted opening of the gating helix in the Sec14 domain. It has also been suggested that the water-exposed surface of the beta protrusion might serve as a docking site for a binding partner, which could, in turn, regulate the process [8,14]. Hitherto, the only characterized functions for the Sec14-PH have been in protein–protein interaction. In fact, Sec14-PH has been characterized in vitro and in vivo as the interacting partner of the endogenous opioid μ- (MORs) receptors activated Gβγ subunits, which are able to inhibit NF1 RasGAP function [15]. The PH module was reported to drive neurofibromin interaction with the serotonin 5-hydroxytryptamine 6 (5-HT_6_) receptor in order to regulate the basal activity of the receptor itself [16].

Based on the difficulty in expressing the protein, the structures of only three mutant Sec14-PH domains have been solved: a missense mutant (I1584V), a deletion mutant (K1750Δ), and a tandem repeat mutant (TD). None of these mutants show any dramatic effect on the global fold of the module, but only minor local changes [8]. The I1584V mutant is structurally identical to the wild type and exhibits the same thermal denaturation behavior. The K1750Δ carries a deletion in the lid-lock protrusion resulting in a local backbone deviation and a flip in the orientation of the side chains of a few subsequent residues. Compared with the wt and the I1584V, the K1750Δ mutant showed a lower denaturation temperature. The TD mutant, carrying a duplication in the linker between the Sec14 and the PH subdomains resulting in a longer loop that could not be solved by the X-ray data, shows a thermal behavior similar to the K1750Δ mutant.

To date, the concerted motion of the lid-lock and the lid helix is the proposed model for the lipid exchange function of this module. Starting from crystal structures, we performed molecular dynamics simulations to compare the dynamics of the wild type with the K1750Δ mutant, which revealed that the lid-lock is fundamental for structural integrality of the bipartite Sec14-PH module and is not prone to any movement, allowing the opening of Sec14 lipid cage according to the so long proposed models. Instead, a completely different motion is observed when lid lock stability is affected, allowing the two submodules to slide along each other with the concomitant opening of the lipid cage, suggesting an alternative mechanism for the reported lipid exchange activity of the NF1 Sec14-PH domain.

## 2. Results

### 2.1. PH Portion of Sec14-PH Domain Is Independently Stable and Shows Scarce Flexibility

Expression of an isolated wt neurofibromin PH domain has been shown to rescue 5-HT6 serotonin receptor-operated cAMP signaling in mutant cells carrying specific NF1-associated mutations in the PH sequence [16]. We then postulated that it could retain structural integrity, albeit in the absence of the Sec14 moiety. Preliminary simulations at 300 K on the entire Sec14-PH module, for both wt and K1750Δ mutant (data not shown), did not reveal any of the hypothesized functional motions in the PH domain.

Molecular dynamics simulations were then performed for a total of 1 μs each on the wt and the K1750Δ isolated PH domain in order to evaluate the effect of K1750 deletion in the lid-lock protrusion (alias lid-lock loop) on the intrinsic motions of the isolated PH domain at 300 K.

**Figure 1:**
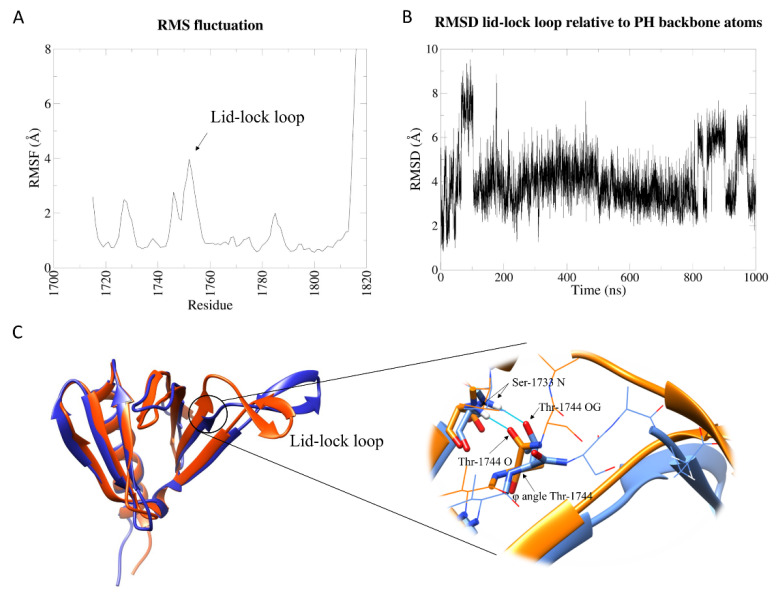
Analysis of the wt isolated PH domain MD simulation. (**A**) Root-mean-square fluctuation (RMSF) of backbone atoms of the PH domain. (**B**) Root-mean-square deviation (RMSD) of the backbone atoms of the lid-lock loop after superposition of the entire PH domain backbone atoms. (**C**) On the left, cluster 1 (orange) and cluster 2 (blue) centroids structures were superimposed. The two structures differ by a conformational change at the N-terminal part of the lid-lock loop, resulting in a left–right swing of the loop; on the right, zoom on the structural differences between cluster 1 and cluster 2 structures. The φ angle of T144 is rotated in cluster 2 structure, resulting in changing the direction of the loop. The carbonyl oxygen of T1744 is substituted by the hydroxyl oxygen (OG) of T1744 sidechain in the H-bond with S1733.

Except for the C-terminal tail, the lid-lock beta protrusion is the most flexible region (Figure 1A) in the PH portion of the Sec14-PH domain; nevertheless, it undergoes very limited motions. To track the position of the lid-lock loop relative to the core, we calculated the root-mean-square deviation (RMSD) of the backbone atoms of the loop throughout the simulation after eliminating the roto-translational motions. The protein behaves as a two-state system: a dominant conformation at RMSD of about 4 Å from the starting structure alternates with another one with an RMSD of 6/8 Å (Figure 1B). A cluster analysis based on the loop RMSD relative to the whole protein backbone (with a cut-off of 2.5 Å) elicited a series of representative snapshots (cluster centroids) of the simulation. Figure S1 and Table S1 show the distribution and numerousness of the 25 resulting clusters. The first five clusters cover 92% of the structures sampled by the simulation, and therefore, their centroids capture the main conformations of the protein. Cluster 1 (70% of total frames) dominates the trajectory and alternates with cluster 2 (9% representativeness), which is enriched when the RMSD is higher. Centroids for these two clusters can be considered the models for state 1 and state 2, respectively (Figure 1C). These two states differ in the conformation of the N-terminal region of the lid-lock loop. In cluster 1, similarly to the crystal structure (as shown in Figure S2), the hydrogen bond between backbone NH of A1746 and backbone carbonyl O of K1731 and the hydrogen bond between backbone NH of S1733 and backbone carbonyl O of Thr1744 stabilize the lid-lock conformation. In cluster 2, the former bond is broken, and the second is substituted by the H-bond between NH of S1733 with the sidechain hydroxyl OH of T1744. This conformational change is due to a rotation in the φ angle of T1744 and results in a slight rotation of the loop towards the outside (Figure 1C, see detail). Nevertheless, none of these conformations could allow the motion required to accommodate the proposed lid helix opening in the Sec14 lipid cage.

Hydrophobic contacts and two specific hydrogen bonds between the lid-lock loop and the lid helix in the Sec14 domain are lost in the isolated PH domain, resulting in a loop slightly rotated in cluster 1, suggesting that the Sec14 portion is involved in stabilizing the conformation of the loop observed in the Sec14-PH crystal. The slight swing of the lid-lock loop represents the whole intrinsic dynamics of the isolated PH domain, indicating generally scarce flexibility in this portion of NF1 protein.

**Figure 2:**
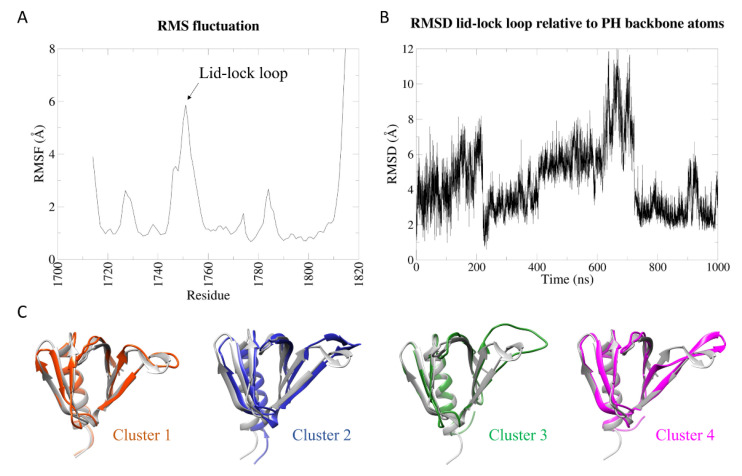
Analysis of the K1750Δ isolated PH domain MD simulation. (**A**) Root-mean-square fluctuation (RMSF) of backbone atoms of the PH domain. (**B**) Root-mean-square deviation (RMSD) of the backbone atoms of the lid-lock loop after superposition of the entire PH domain backbone atoms. (**C**) Centroids of the first four clusters (colored) were aligned against the crystal structure (grey). Cluster 1 and cluster 4 resemble state 1 and state 2 of the wt and the left-right swing due to conformational change in the beta strand before the loop. Cluster 2 and cluster 3 show the “up motion” of the loop, with the flip in the bending orientation (cluster 3) that could not be observed in the wt.

Differently from the wild-type protein, the K1750Δ mutant shows higher intrinsic flexibility in the lid-lock loop in the isolated PH domain (Figure 2A). According to the crystal structure of the mutant [8], the K1750 deletion only causes local structural changes. Due to the shortening of the loop, A1746 in the mutant is not structurally aligned with A1746 in the wt due to a modification in the backbone shape, causing the weakening of the H-bond between backbone NH of A1746 and backbone carbonyl O of K1731 (see Figure S2 for a comparison to the wild-type structure). As mentioned, this interaction was found to stabilize state 1 in the wt simulation. Furthermore, starting from E1747, the structural alignment is recovered, but the sequences are shifted by one position up to wt K1750 and mutant T1749. Taken together, these two structural differences observed in the crystal structures of wt and mutant PH domain, higher flexibility of the mutant is expected, especially in the absence of the Sec14 domain.

While retaining the general compactness of the domain core, in the mutant, the beta-protrusion explores a higher number of different conformations compared to the wild type, some of them compatible with the formerly invoked open configuration [14], as suggested by the lid-lock loop RMSD relative to the whole PH domain (Figure 2B). Cluster analysis with a 4 Å cut-off reveals that many different conformations are explored during the simulation (Figure 2C and Figure S3 and Table S2). As for the wt, a dominating cluster 1 (64% of the total) resembles the crystal structure. Cluster 4 represents a structure analogous to wt state 2, with the same conformational change in the N-terminal region of the lid-lock loop, but the dynamics of the mutant protein cannot be described by a two-state model. Clusters 2 and 3 are the largest among the clusters that explore conformations that could not be observed in the wt protein dynamics. In particular, the most dramatic conformational change is shown in cluster 3, which is mainly explored between 600 ns and 700 ns. In the centroid structure, the lid-lock loop moves upwards and flips its bending orientation.

### 2.2. Dynamic Behaviour of the Whole Wild-Type Sec14-PH Domain

Due to the interactions between the lid-lock loop with the Sec14 upper lid helix, none of the intrinsic dynamics similar to the ones observed for the isolated PH domain could be observed in the first 1 μs-MD simulation at 300 K for the whole Sec14-PH domain. In order to explore more possible states for the Sec14-PH domain, we performed a series of simulations at progressively higher temperatures (300 K, 330 K, 340 K, 360 K, 380 K, and 400 K).

**Figure 3:**
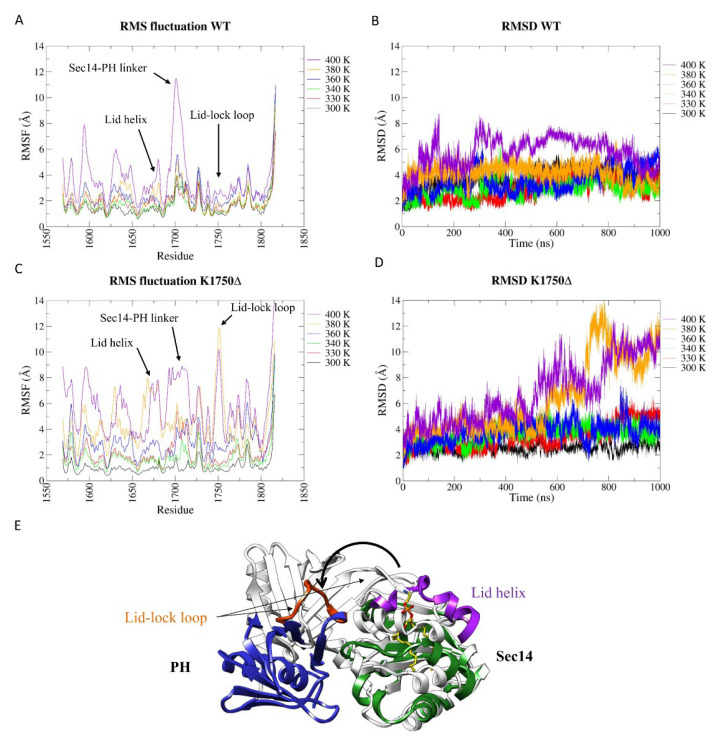
Analysis of MD simulations at increasing temperatures. (**A**) Root-mean-square fluctuation (RMSF) and (**B**) root-mean-square deviation (RMSD) of wt Sec14-PH domain. (**C**) Root-mean-square fluctuation (RMSF) and (**D**) root-mean-square deviation (RMSD) of K1750Δ mutant Sec14-PH domain. (**E**) A representative frame from the 380 K simulation of the mutant Sec14-PH domain. The lid-lock loop lost its secondary structure, and consequently, the PH portion detached from the Sec14 moiety.

The root-mean-square fluctuation (RMSF) of the wt Sec14-PH domain shows increased flexibility within specific regions when the temperature rises to 400 K (Figure 3A), in line with a loss in secondary structure content in those regions corresponding to peaks in RMSF. Accordingly, the RMSD shows a similar pattern throughout the simulation from 300 K to 380 K (Figure 3B), but at 400 K, it seems to deviate more from the starting conformation. The most affected part was the linker peptide between Sec14 and PH subdomains (aa 1700–1714). Overall, the PH module (aa 1715–1816) shows to retain its structure, while Sec14 appears more susceptible to temperature: in fact, increased flexibility can be observed in the helices that constitute the lipid cage. The lid-lock loop did not show the same flexibility observed in the simulations of the isolated PH domain. In the context of the whole module, the lid-lock loop appears to be one of the more stable regions due to the stabilizing interaction with the Sec14 upper lid helix.

### 2.3. The K1750Δ Mutation Weakens the Interconnection between the Two Portions of the Bipartite Sec14-PH Domain

A completely different behavior was observed during the MD simulation for the K1750Δ mutant Sec14-PH domain. A dramatic increase in flexibility was observed already at 380 K and furthermore at 400 K, as can be observed in the RMSF graphs (Figure 3C). Differently from the wild type, the RMSD plots at both 380 K and 400 K show that the tertiary structure dramatically deviated from the starting conformation (crystal structure) at around 500 ns (Figure 3D). Interestingly, in both the simulations at these temperatures, the lid-lock loop has a pick in flexibility (Figure 3C). These results agree with experimental thermal denaturation data reported in the literature [8], describing a CD signal, suggesting a lower melting temperature for the mutant relative to the wt. The analysis of the secondary structure percentage in the structures explored during the 400 K simulations confirms that the K1750Δ mutant Sec14-PH loses more secondary structures than the wild-type (Figure S4) but conserves the same level of secondary structure as the wt at the lower temperatures.

The analysis of the structures sampled in the second half of the simulations performed at 380 K and 400 K revealed that the lid-lock loop exhibits the same dynamic behavior as in the isolated PH domain at room temperature, with a flip in the bending direction (Figure 3E). This resulted in the loss of contacts between Sec14 and PH portions and, eventually, in the physical separation between the two subdomains, revealing that the lid-lock contact with the lid helix is fundamental for the structural integrity of the domain and that higher flexibility of the lid lock does not induce or allow the lid helix opening, but disrupts the domain.

### 2.4. Opening of the Lipid Pocket

**Figure 4:**
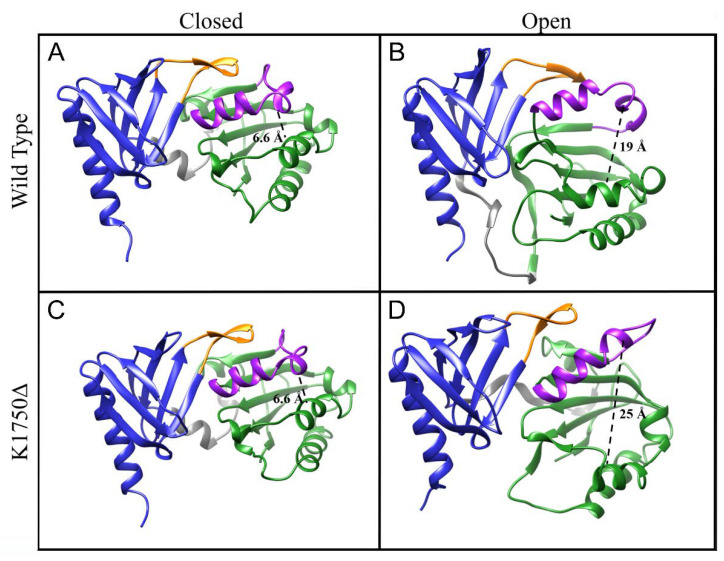
Comparison of the closed and open conformation for the Sec14 domain of neurofibromin. The distance between the α-carbons of S1639 and L1675 is shown with a dashed line. Those frames were chosen from trajectories at 400 K for wt (**A**,**B**) or K1750Δ (**C**,**D**) in which two conditions were satisfied: the maintenance of the secondary structure content, especially in the helices of the lipid pocket, and an S1639Cα–L1675Cα distance compatible with an open conformation (for **B**,**D**), using as a reference the 23 Å in the yeast Sec14p.

Ryan and co-workers [11] used molecular dynamics to study the closing of the lid helix in the yeast Sec14p, which was crystallized in the open conformation (PDB 1AUA [10]). They monitored the distance between the alpha-carbon of R195 and F231 (on the lid helix). In the crystal structure, that distance was 23 Å, and in their simulation, it dropped to around 10 Å in agreement with a closed conformation. A closed conformation was crystallized later for the yeast Sec14 homolog protein (Sfh1, PDB 3B74), revealing a distance between K197 and F233 (corresponding to R195 and F231 on Sec14p, respectively) of 6.9 Å. Structural alignment of NF1-Sec14 on Sfh1 shows that the corresponding residues to R195/K197 and F231/F233 are S1639 (on α3 helix) and L1675 (on the lid helix), respectively, whose alpha-carbon distance is 6.6 Å (Figure 4A). Inspired by this previous work, we applied the same approach to neurofibromin, analyzing the results of our simulations to check for a structure compatible with an open conformation and suitable for lipid exchange.

Figure S5 shows the distributions of the distance between S1639Cα and L1675Cα for the wt. From 300 K to 360 K, we can see a sharp distribution with a peak at 7.5 Å, close to the 6.6 Å value of the crystal structure (Figure 4A), in agreement with the overall stability suggested by RMSD and RMSF graphs. As the temperature rises to 380 K and 400 K, the pick shifts towards around 12 Å with more spread distributions.

At 400 K, the S1639Cα–L1675Cα distance reaches values over 23 Å, but they are mainly associated with loss in secondary structures that occur at 400 K, causing large distortions in the internal coordinate of the protein. In the wt, those frames in which secondary structures are preserved never show an S1639Cα--L1675Cα distance over 20 Å (Figure S5). A representative structure for a putative open conformation with a 19 Å opening is shown in Figure 4B. The lid-lock loop is slightly shifted relative to its position in the crystal structure and moves in a fashion such as that observed in the PH domain alone, while the Sec14 lipid pocket opens up by the relative rotation of the two submodules, where the lid helix moves together with the PH moiety rather than with the Sec14 module.

The mutant protein follows the same trend as the wt regarding the opening of the lipid cage. Up to 360 K, there is no significant deviation from the starting value (Figure 4C and Figure S6). At 380 K and 400 K, the pattern is similar to the wt one, with the distributions getting broader. As shown earlier, the mutant domain undergoes a dramatic conformational change already at 380 K, with the lid-lock loop opening and the PH domain losing contact with Sec14. This, in turn, impinges on the stability of many helical segments of the Sec14, which unfold to coiled coil. Nevertheless, before the conformational transition occurred, it was possible to observe and analyze frames that satisfied the helical component of the lipid cage and an S1639Cα–L1675Cα distance compatible with a hypothesized open conformation. A representative frame in which such distance is 25 Å is shown in Figure 4D. We can see that, also for the mutant protein, the lid-lock loop does not need to alter its internal conformation but is rather moved away from its original position, together with the lid helix, by a rigid body rotation of the PH domain.

## 3. Discussion

The molecular bases of the pathogenesis of neurofibromatosis have been thoroughly investigated in recent years, thanks to the availability of several animal models [17]. Unfortunately, no progress was made in understanding the molecular mechanisms regulating NF1 functions, including RasGAP activity, with the outcome that effective therapies are still unavailable. This is mostly related to the intrinsic difficulty in NF1 manipulation due to the huge size and the impossibility of expressing and purifying full-length or large fragments in *E. coli* due to the incompatibility of the full cDNA with this host [7]. Structural data on NF1 protein portions and full protein are consequently scarce and precious [18,19].

Scheffzeck and co-workers [8] applied circular dichroism to investigate the thermal stability of both wt and a series of mutants in the Sec14-PH domain and concluded that the K1750Δ mutant loses its structure at a lower temperature relative to the wt. Our results confirm that, although the K1750Δ mutant Sec14-PH domain is able to adopt a conformation similar to the wild-type domain, which is proved by structural data and confirmed by the analyses of the conformations explored during the simulation, the mutation still provokes increased flexibility in the lid-lock loop, both in the isolated PH domain (Figure 2A,B) and in the whole Sec14-PH domain (Figure 3C,D). This flexibility could impinge on the overall stability of the Sec14-PH domain, both in terms of secondary structure loss and, most importantly, in terms of structural dependence of the two subdomains. In fact, while the wt PH domain is characterized by scarce flexibility both alone and within the bipartite domain (Figure 1A and Figure 3A), the Sec14 moiety is characterized by higher flexibility, which matches the flexibility of the lid-lock loop in the mutant structure (Figure 3A,B). The loss of contact between the PH lid-lock loop and the Sec14 lid helix has consequences on the connection of the lid helix and impinges on the overall stability of the Sec14 lipid cage. In no case, either in the wild type or in the more flexible K1750Δ mutant, any motion was observed that could lead to the lid helix opening as previously proposed. Whenever the lid lock loses contact with the lid helix, the connection between the two moieties of the Sec14-PH domain is also lost.

Taken together, our results suggest that the structural integrity of the Sec14-PH domain requires a tight interconnection within the two modules at the lid-lock level and that its loss damages the structural integrity of the Sec14 moiety as well. It is worth noting that a pathogenic mutant with a tandem repeat insertion in the linker between Sec14 and PH is known to possess a defect in structural stability similar to the K1750Δ mutant [8]. Given our results, it is possible that altering the flexibility of this linker may as well affect the tight structural interconnection between Sec14 and PH domain and so the overall stability of the Sec14 module.

Finally, the results of our simulations suggest that the main role of the lid-lock loop would be to retain the tight association of the double module rather than to modulate the putative lipid exchange function, as previously proposed [14]. As a matter of fact, the neurofibromin Sec14 subdomain was shown to exert lipid exchange activity in vitro [13], but no biological function was hitherto attributed to this activity. In contrast, physiological protein–protein functions were reported for either the Sec14-PH domain or the PH subdomain itself [15,16], which would be affected by a loss in the overall Sec14-PH domain conformation sturdiness. The observed lipid exchange activity would require the opening of the Sec14 module, but this could rather be achieved without disturbing the connection of the lid-lock with the lid helix, with a joint motion of the whole PH domain together with the lid helix with respect to the rest of Sec14 lipid cage. Based on the analysis of the structures with an open conformation of the Sec14 lipid pocket observed during the MD simulations of both wt and K1750Δ mutant neurofibromin Sec14-PH domain, we propose that a different mechanism for the opening of the Sec14 lipid cage could exist. This alternative mechanism would not require the lifting of the lid-lock to allow the movement of the lid helix as previously proposed [14], but it would rather happen by a rotation of the two submodules where the lid helix maintains its connections to the lid-lock and follows the PH domain in its slide along with the Sec14 moiety (see Video S1 and S2 for a representation).

## 4. Materials and Methods

### 4.1. Systems Setup

The coordinates of the neurofibromin Sec14-PH domain were obtained from the Protein Data Bank [20] (PDB ID: 2E2X for the wild type [13] and PDB ID: 3PG7 for the K1750Δ mutant [8]). Some initial residues were removed so that both WT and mutant started at L1569. Starting point coordinates for the PH domain alone were obtained from the correspondent Sec14-PH structure by removing the Sec14 moiety (from 1569 to 1714). For all systems, N-terminus was then capped with an acetyl (ACE) group, and the C-terminus was capped with a N-methyl amide (NME) group. All systems setup and subsequent simulations were performed with Gromacs 2018.2 [21]. PH domains were solvated in a cubic box with dimensions of 73.66 × 73.66 × 73.66 Å, filled with CHARMM-TIP3P water molecules [22], and neutralized with 150 mM NaCl. Sec14-PH proteins were solvated in a cubic box with dimensions of 92.06, 92.06, and 92.06 Å, filled with CHARMM-TIP3P water molecules, and neutralized with 150 mM NaCl.

### 4.2. Molecular Dynamics Simulations and Analysis

All MD simulations were performed with GROMACS 2018.2 and with CHARMM36m-nov2018 forcefield [23] for protein and the Charmm General Forcefield (CGenFF) [24] for the lipid molecule. For PH domains, a single simulation at 300 K was performed for both the wt and the mutant. For the Sec14-PH domains, six independent simulations, each with a different temperature, were performed for both the wt and the mutant (300 K, 330 K, 340 K, 360 K, 380 K, and 400 K). Before all the production simulations, the systems were minimized with the steepest descent method until maximum force went below 1 kJ/mol/nm. The minimization was followed by a 1 ns NVT equilibration phase at the target temperature with the V-rescale algorithm [25] to stabilize the temperature and a 1 ns NPT equilibration phase with the Berendsen [26] algorithm to stabilize the pressure (1 atm). Both equilibration phases were performed with 1 fs time-steps and with position restraints on protein- and lipid-heavy atoms. The production runs continued in the NPT ensemble without position restraints for 1 μs, with the V-rescale method for stabilizing the temperature and the Parrinello–Rahman barostat [27] for the pressure (1 atm). Snapshots of the coordinates were written out every 100 ps. A 2-fs time-step was used throughout the production simulations. For both short-range electrostatics and Lennard–Jones, a 12 Å cut-off was applied, in the latter case with a force switch at 10 Å. Long-range electrostatic interactions were calculated with the particle-mesh Ewald method [28,29] with a grid space of 12 Å, and bonds involving hydrogen atoms were constrained using the LINCS algorithm [30]. Periodic boundary conditions were applied. All computation was performed on the CINECA Marconi supercomputer.

All subsequent analyses (RMSD, RMSF, cluster analysis, principal component analysis, distances, and secondary structures) were performed with Gromacs 2018.2 on the 10,000 frames trajectories coming out of the simulations. Graphs were plotted with Xmgrace. All protein images and videos were rendered with Ucsf Chimera 1.14 [31].

## Data Availability

Not applicable.

## Supplementary Materials

The following supporting information can be downloaded at: https://www.mdpi.com/article/10.3390/ijms23105707/s1.
