# Supplementary material for: Molecular Dynamics Simulations Reveal Structural Interconnections within Sec14-PH Bipartite Domain from Human Neurofibromin
Source: Int J Mol Sci. 2022 May 20;23(10):5707. doi: 10.3390/ijms23105707 (PMC9147397; doi:10.3390/ijms23105707)
Supplement: Supplementary file 1 [file ijms-23-05707-s001.zip › ijms-1727246-supplementary.pdf]

Supplementary information

Supplementary Tables.

Table S1. Probability of cluster occurrence for the wild type isolated PH domain. Clusters were defined based on backbone atoms RMSD with a cutoff of 2.5 Å.

Cluster	Cluster occurrence (%)
1	69.97
2	9.36
3	6.67
4	4.66
5	1.77
6	1.49
7	1.38
8	1.26
9	1.25
10	0.52
11	0.29
12	0.28
13	0.22
14	0.19
15	0.18
16	0.17
17	0.11
18	0.06
19	0.05
20	0.03
21	0.03
22	0.02
23	0.02
24	0.01
25	0.01

Table S2. Probability of cluster occurrence for the K1750Δ isolated PH domain. Clusters were defined based on backbone atoms RMSD with a cutoff of 4 Å.

Cluster	Cluster occurrence (%)
1	63.71
2	8.42
3	7.07
4	5.86
5	3.47
6	2.45
7	2.07
8	2.03
9	1.16
10	0.99
11	0.53
12	0.43
13	0.32
14	0.26
15	0.21
16	0.17
17	0.15
18	0.14
19	0.12
20	0.10
21	0.08
22	0.05
23	0.05
24	0.03
25	0.03
26	0.03
27	0.03
28	0.02
29	0.01
30	0.01

Supplementary Figures

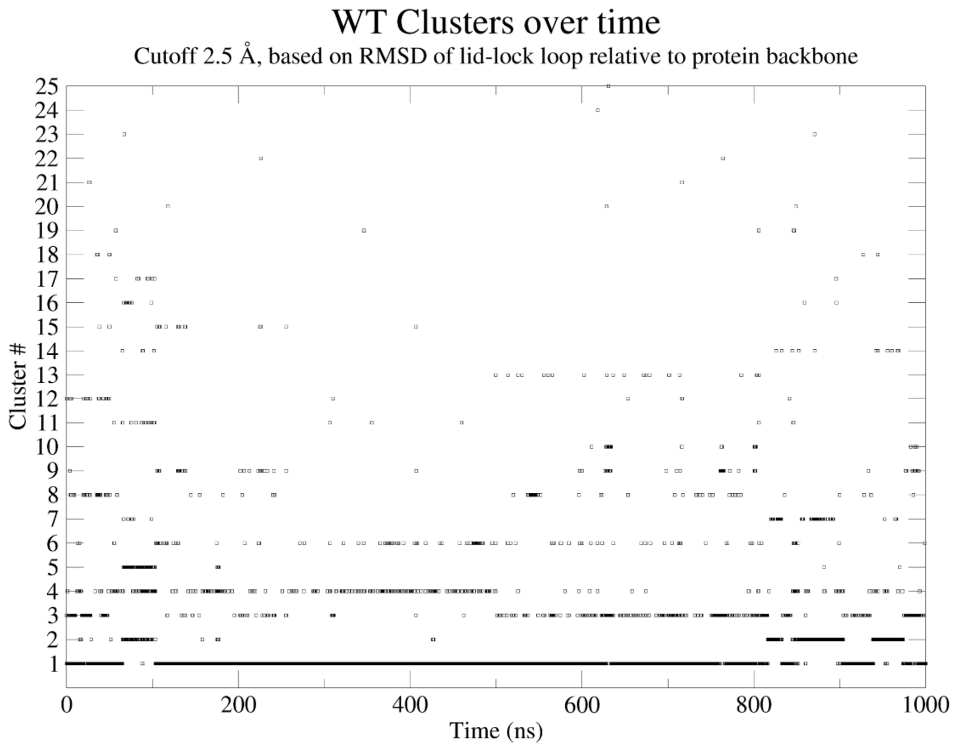

Figure S1. Wild-type MD simulation clusters distribution over the trajectory

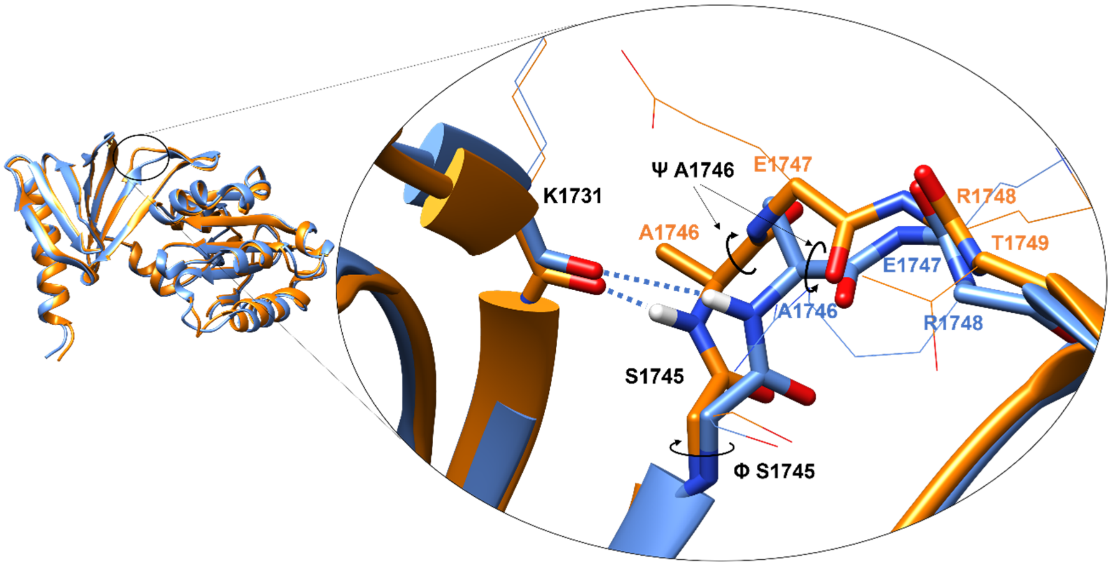

Figure S2: Superposition of the wild-type and K1750Δ mutant Sec14PH domain structures. Despite the deletion being located at residue 1750, the lid-lock backbone of K1750Δ (blue) gets distorted when compared to the WT (red) starting from the residue S1745. In fact, a different φ dihedral (-68° in the WT vs -104° in the mutant) induces the A1746 main chain NH of the mutant to move further from the K1731 main chain carbonyl oxygen with respect to the wild-type structure. This causes the H—O distance to stretch from 1.638 Å in the WT to 2.605 Å in the mutant and the O—H—N angle to tighten from 172° to 152°, with an overall weakening of the H-bond. The subsequent rotation change in the A1746 ψ dihedral from -19° in the WT to 173° in the mutant allows the recovery of the structural alignment of the backbone to the wild-type structure and the correct fold of the lid-lock loop.

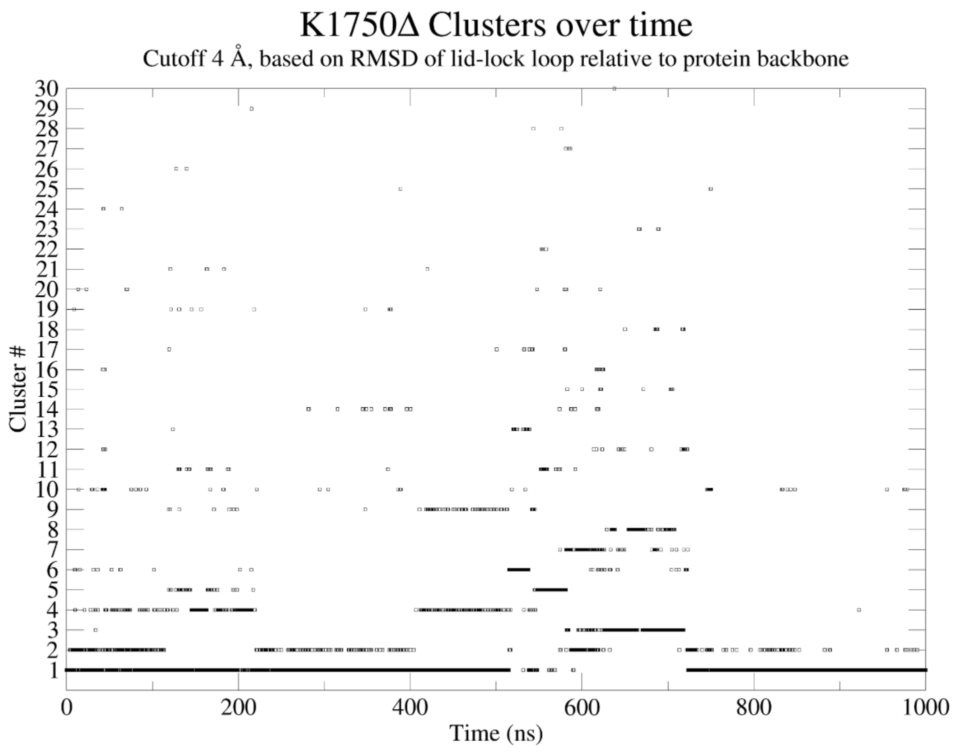

Figure S3. K1750Δ mutant MD simulation clusters distribution over the trajectory.

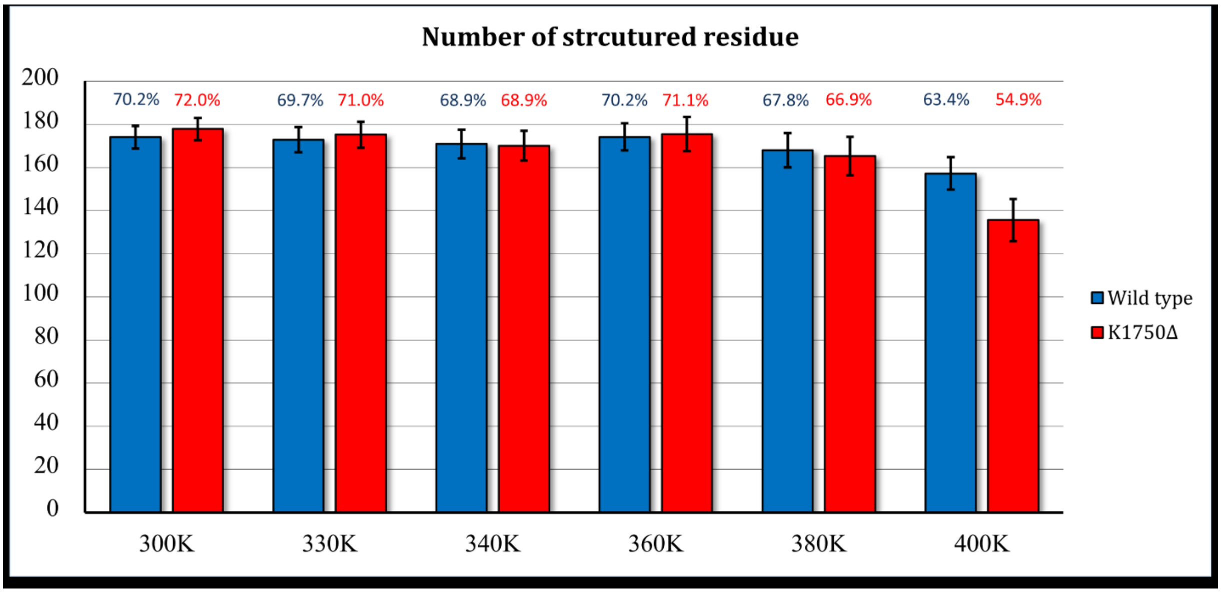

Figure S4. Percentage of secondary structure at different temperatures, for wt and mutant Sec14-PH.

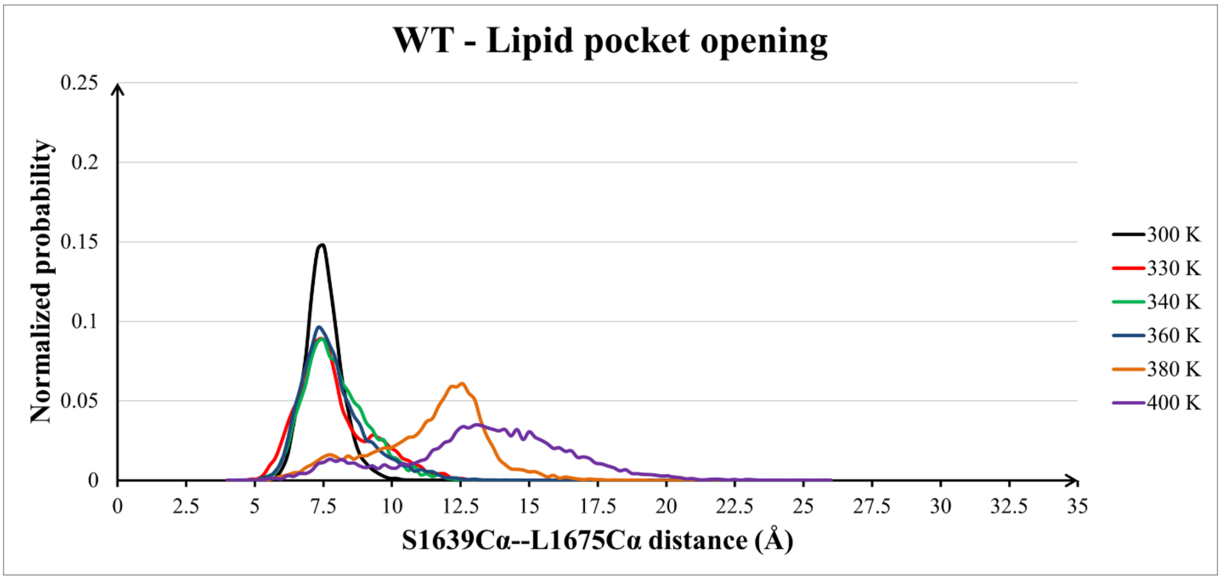

Figure S5. Distributions of the distance between the Cα of S1639 and L1675 as a probe for the opening of the lipid pocket in the wild type Sec1-PH.

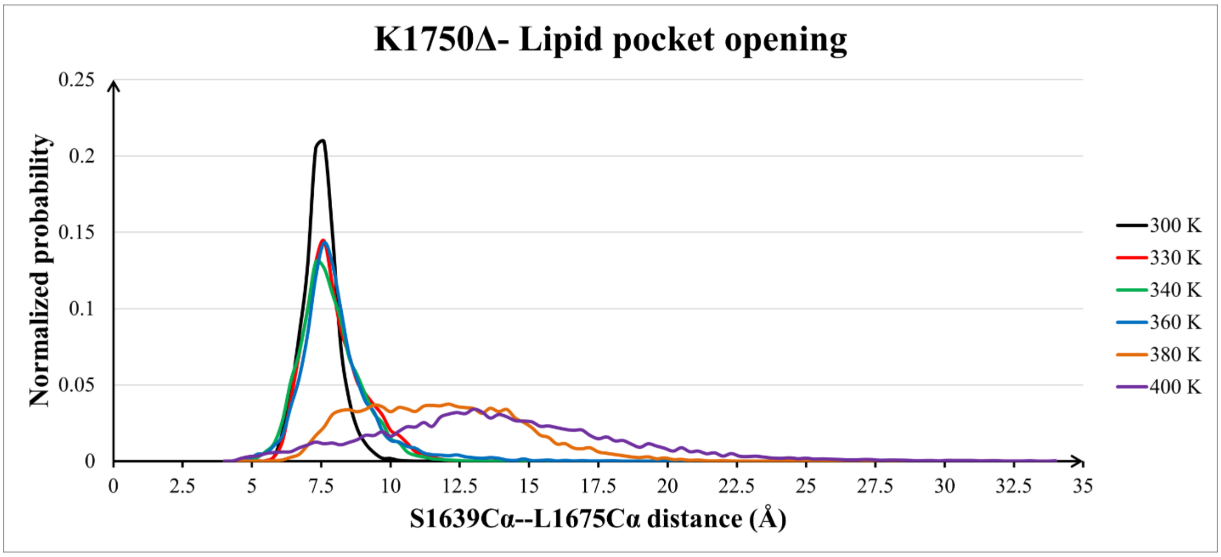

Figure S6. Distributions of the distance between the Cα of S1639 and L1675 as a probe for the opening of the lipid pocket in the K1750Δ mutant Sec1-PH.

Supplementary Movies.

Video S1. Proposed model for lipid pocket opening in the wild-type Sec14-PH domain. The trajectory is an interpolation between the crystal structure and a frame at 400K showing an open lipid cage conformation while preserving the secondary structures.

Video S2. Proposed model for lipid pocket opening in the K1750Δ mutant Sec14-PH domain. The trajectory is an interpolation between the crystal structure and a frame at 400K showing an open lipid cage conformation while preserving the secondary structures.
